# Parents and Mobile Devices, from Theory to Practice: Comparison between Perception and Attitudes to 0–5 Year Old Children’s Use

**DOI:** 10.3390/ijerph18073440

**Published:** 2021-03-26

**Authors:** Loredana Covolo, Daniela Zaniboni, Jacopo Roncali, Valentina Mapelli, Elisabetta Ceretti, Umberto Gelatti

**Affiliations:** 1 Department of Medical and Surgical Specialties, Radiological Sciences and Public Health, Section of Public Health and Human Sciences, University of Brescia, 25123 Brescia, Italy; elisabetta.ceretti1@unibs.it (E.C.); umberto.gelatti@unibs.it (U.G.); 2 Post-Graduate School of Public Health, University of Brescia, 25123 Brescia, Italy; d.zaniboni001@unibs.it (D.Z.); j.roncali@unibs.it (J.R.); 3 Degree Course in Health Assistance, University of Brescia, 25123 Brescia, Italy; valentina_mp1995@hotmail.it

**Keywords:** children’s health, digital health, mobile device, screen-time, parents, children, risk

## Abstract

Pediatrics associations recommend avoiding the use of mobile devices (MDs) in children aged 0–2 years and limiting them to one hour per day for children aged 3–5 years. This study aimed to explore public risk perception on MDs use by children aged 0–5 years and attitudes of parents to children’s use. Participants were recruited on a voluntary basis by social media using a self-administered survey. The study included 3115 participants, most of whom were parents of children aged under 5 years (*n* = 1901; 61%). Most of the respondents (74%) considered that MDs use could be harmful for children’s health. The awareness on a correct use did not always translate into avoiding MDs use as recommended, especially in parents of children aged 0–2 years. Half of the sample (54%) received or sought information on risks related to MDs use. The most consulted information sources were the web (78%), and pediatricians in only 22% of cases. Understanding the determinants of parents’ risk perception and behavior is necessary to design effective family-based interventions in order to guide parents to a moderate and more careful use of MDs by their children. To do so, public health actions must aim to strengthen awareness about conscious use of MDs.

## 1. Introduction

The widespread diffusion of MDs (mobile devices) is greatly encouraged by their characteristics: easily transportable, pocket sized, mainly low cost, and containing multiple interactive applications. In 2020 “We are social” data showed that there were 49.48 million mobile users in Italy (82%) who spend, daily, 1 h and 57 min on social networks, on average [1].

Children are nowadays defined as ‘digital natives’ as they grow up surrounded by these technologies. Overall, literature has shown how children use more smartphones (40.8%) than television (39.6%) and tablet/computer (19.6%). In the recent few years, there has been a marked increase in mobile phone use by children, including those aged under 2 years [2,3,4,5], and time spent in front of MDs, that is ‘screen-time’, is becoming a not negligible part of their life-time [6].

The onset age of use of these MDs seems to be gradually lower over time, and up to half of children aged 0–3 years use at least one MD for an average of about 2–5 h per day [5,7,8,9].

A study conducted in Italy by “CBS Onlus—Center for Child Health Onlus” [10] has shown that 20% of children use a smartphone for the first time during the first year of life. Eighty percent of children aged 3–5 years old are able to use their parent’s smartphone. Parents often use media as pacifiers, giving MDs to their child to keep them calm during the first (30%) and the second (70%) year of life. It has also been shown that parents allow the use of MDs to their children to keep them busy while carrying out domestic activities, to facilitate their sleep and to keep them calm rather than simply consoling the child in times of crying [3,7,11].

The situation where time spent on MD is without the presence of an adult could be more problematic as there is no parental control. Of note is that the parent–child relationship is very crucial in a child’s cognitive development, especially in the early stages of life [12,13]. Generally, parent and child co-use of MDs is highest for children younger than 2 years and decreases as the child ages [10,14].

Studying MDs effects, especially in the first years of life, arises from the need to preserve a life-period during which physical, psychomotor, social-emotional, mental and language development are substantially completed, personality is shaped, and the child is continuously changing [12]. Habits of childhood are reflected in adolescence and adulthood, and therefore, this underlines the importance of analyzing, and therefore regulating, the use of MDs at an early age.

The literature is very rich in studies regarding exposure to screens such as television and computers. Only in recent years have we witnessed the need for studies investigating health effects regarding MDs use in children, and even more recently, the literature has focused on use in young children [15]. A recent systematic review demonstrated that screen-time was associated with obesity, high depressive symptoms and poorer quality of life, in addition to poorer educational attainments and negative cognitive development in younger children. Moreover, there is evidence that supports the association between poor sleep outcomes and all forms of screen-time, including mobile phone, video, computer and television [16]. The hypothesized mechanisms of damage are direct and indirect. Direct mechanisms can be linked to contents watched on screens, blue screen light on sleep patterns and concentration [8,17,18]. Moreover, eye and vision problems were associated with mobile phone use, particularly at pediatric age [14,19]. Indirect mechanisms lie in time spent sitting (e.g., sedentary lifestyle) and loneliness (e.g., social isolation) [6].

Furthermore, children might develop an addiction to MDs, therefore considering them indispensable for their life [7]. In fact, the diffusion of risks related to problematic smartphone use in children and particularly adolescents is a matter of concern [20].

Regarding possible health benefits from screen-time, the results of a systematic review has shown that, in case of interactive MDs, there are learning and playing opportunities, as well as face-to-face connection with distant family and friends [14]. Otherwise, a more recent systematic review found no consistent evidence on health benefits and screen-time [6].

Even though not conclusive, the evidence so far is enough to raise concerns, so that scientific societies have tried to draw up guidelines that regulate use of these MDs in time, accessibility and supervision. The Italian Society of Pediatrics (SIP) [21], in line with other international societies [22,23], suggests that MDs exposure during childhood should be thus regulated: no use in children under the age of 2, during meals, 1 h before bedtime, in case of violent and frenetic programs and avoid use of MDs as ‘pacifiers’, to keep children quiet in public places. Then, limit exposure in children aged 2–5 years to a maximum of 1 h per day, and in children aged 5–8 years to a maximum of 2 h, and prefer high-quality programs and encourage vision in the presence of an adult. The SIP strengthens the parental “good model”, given that children are great imitators.

Attention has also been paid to the type of content of the MDs. In fact, the Royal College of Paediatrics and Child Health, in their 2019 report, claim that children can potentially be exposed to inappropriate material or interactions, as they can be in the non-digital world (e.g., bullying or violence). Latest revisions of guidelines offer to parents a way to understand when the use is excessive and to build limits [24]. They underline that the effect of screen time depends strongly on family context [9,25,26]. In fact, studies focused on adults’ screen-time behavior and screen-time restrictions for children showed that adults with lower screen time and perceived severity of smartphone addiction more effectively exercise their role of control on the duration of their children’s screen time [9,26,27,28]. Most of these studies involved children in primary school. Schoeppe et al. [27] also pointed out that despite the fact that most adults thought it was appropriate to limit children’s screen time as recommended, few of them adhered themselves to this recommendation. In a similar way, it occurs that parents are aware about healthy behavior to be adopted to prevent overweight and obesity in their children, but they do not put their knowledge into practice [29,30].

To our knowledge, few studies have investigated the perception of the public on risks related to MDs use by children, especially young children. The aim of this study was firstly to explore the opinion of adults regarding the use of MDs by 0–5-year-old children. Secondly, on the assumption that parents of younger children are generally more apprehensive about health risks to their children, the aim of the study was also to understand if being parents of younger children increased risk perception compared to non-parents and parents of older children. Finally, the aim was to investigate the relationship between risk perception and parental attitudes to MDs use by their children.

## 2. Materials and Methods

### 2.1. Participants and Procedures

A cross-sectional study was carried out in Italy from June to October 2018. Data were collected anonymously by an online self-administered questionnaire, created ad hoc (Supplementary File S1).

The inclusion criterion was being aged 18 years and more. The questionnaire was designed using LimeSurvey software (LimeSurvey GmbH, Hamburg, Germany) and it was accessible by clicking on a link. It was disseminated by the investigators using social media such as Facebook, Instagram and WhatsApp. Moreover, the questionnaire was distributed through WhatsApp groups and different Facebook groups, including those dealing with children’s health that have thousands of subscribers, which were asked to further disseminate the questionnaire (Snowball sampling).

Before being disclosed, it was administered to a small group of people, about 20, who filled it out, expressing doubts or misunderstandings in order to make changes that would make the questionnaire more understandable and effective.

### 2.2. Measurements

The questionnaire consisted of 3 sections. The first contained questions regarding socio-demographic data (gender, age, educational qualification, employment, country of origin and parental status). The second section included questions on general habits of participants about their use of MDs, specifically smartphone and/or tablets (the number of MDs used per day and time spent on them). Then, participants were asked about their general risk perception of using MDs by children aged 0–5 years and, in order to better evaluate risk perception, they were asked opinions on specific risks and benefits related to MDs use. The third section included questions addressed only to those who declare to be parents of children aged 0–5 years and comprised questions related to the attitudes of parents in relation to the use of MDs by their children.

All participants were asked if they considered that the use of MDs by preschoolers posed a health risk. If participants answered ‘yes’, they were asked to assess the risk giving a score on the basis of a 7-point Likert scale (1 = not harmful and 7 = very much harmful). A higher score indicated a higher level of risk perception.

Participants were asked to indicate the time of MDs use that they thought was correct, respectively for children aged 0–2 years and children aged 3–5 years. The responses were categorized in “zero minutes”, “until 60 min” and “more than 60 min” in both the age groups.

Using SIP guidelines as a reference [21], we defined as “correct”: zero minutes for children aged 0–2 years and one hour maximum for children aged 3–5 years.

Perception about specific risks and benefits was evaluated separately using two sets of questions which included for each item a 7-point Likert scale with 1 “no risk or no benefit” and 7 “high risk or high benefit”. There was also the option “I don’t know”. The scores of the Likert scale were categorized as follows: “no risk” and “no benefit” correspond to score 1, “low risk/benefit” to scores 2, 3, 4 and “high risk/ benefit” to scores 5, 6, 7.

We selected symptoms or diseases as risks and behavioral or social factors as benefits based on recent systematic reviews [14,15]. Among risks we included “developing celiac disease” and “epilepsy”, for which there was no scientific evidence for the association with MDs use. These items referred to diseases known by public and they were included to verify appropriateness of the responses by testing the ability to discriminate a more likely risk from a less realistic one. For the same reason “preventing ADHD (Attention Deficit Hyperactivity Disorder)” was included among possible benefits.

Among benefits, the possibility of learning new words was considered separately for children aged 0–2 years and 3–5 years, to highlight whether the age range of the little ones was considered more to be safeguarded.

Parental attitudes to the use of MDs by their children focused on whether parents allowed the use of MDs, the time use in case of positive response, whether the use was in sharing with an adult, the reasons of use by children and the motivations why parents spontaneously gave the MD to their children.

Finally, sources of information on risks related to MDs use by children were investigated.

### 2.3. Data Analysis

Comparisons between groups were made by using the χ2 test or Fisher’s exact probability test for categorical variables and Student’s parametric t test or the Wilcoxon non-parametric test for continuous variables. A multiple logistic regression model was performed to evaluate the factors associated with the perception that the use of a mobile device by preschoolers was a risk for their health. The answer “yes” versus “No/don’t know” to the question “Does the use of a MD by a preschool child (0–5 years) pose a risk to his health?” was used as an independent variable. A multiple logistic regression model was also performed to evaluate the potential predictors leading parents to allow the MDs use by their children. The answer “yes” versus “No” to the question”, Do you usually let your child use the smartphone and/or tablet?” was used as an independent variable. The covariates to be included into the final models were selected using a stepwise backward selection process, with a univariate *p* < 0.05 as the main criterion. Results were expressed as Odds Ratio (OR) with 95% confidence interval (CI). *p*-value < 0.05 was considered significant for all analyses. Statistical analyses were performed using STATA (Stata Statistical Software: Release 14.0, Stata Corporation, College Station, TX, USA).

## 3. Results

A total of 3115 questionnaires were completed by the participants. They were predominantly female (*n* = 2689; 86%), parents (*n* = 2540; 82%), with a mean age of 37.2 ± 10.5 years.

Of the participants, 36% (*n* = 1106) had a university degree, most of them were employed (*n* = 1738; 56%), 16% (*n* = 499) were housewives and 14% (*n* = 427) were self-employed workers. Almost all participants were Italian (*n* = 2957; 95%). Most of the parents (*n* = 1901; 75%) had children aged 0–5 years (Table 1).

When considering the whole sample, 96% (*n* = 3004) used one or more devices per day, in particular, most of the respondents used only one device on a daily basis (*n* = 2013; 65%), and about a quarter (*n* = 833, 27%) used two devices. The average time of use was 3 ± 2.5 h a day, particularly 3.6 ± 2.6 h for participants who used two MDs or more and 2.9 ± 2.4 h for those who used one MD (*p* < 0.001). Younger people (18–35 years old) reported to use MDs for longer than participants aged more than 35 years (3.6 vs. 2.6 h/day; *p* < 0.001). Woman reported to use MDs for longer than men (3.2 vs. 2.8 h/day; *p* < 0.001). Unemployed people and those with a university degree used MDs for longer than employed people (3.5 vs. 3 h/day; *p* < 0.001). Non-parents reported to use MDs for longer than all parents (3.8 vs. 3 h/day; *p* < 0.001). Parents of children aged 0–5 years spent more time on MDs than parents having older children (3.1 vs. 2.4 h/day; *p* < 0.001).

For the question “In your opinion, does the use of a smartphone and/or tablet by a child (0–5 years) pose a risk to his health?”, 74% (*n* = 2.304) answered it was a health risk. Non-parents (80%) and parents of children aged over 5 years (82%) claimed the existence of a risk more than 0–5-year-old children’s parents (70%) (*p* = 0.001) (Table 2).

Respondents who declared that use of MDs by children under 5 years was a health risk were asked to express it on a scale of 1 (= Not harmful) to 7 (= Very harmful). The majority (76%) indicated a score higher than 5, particularly parents of children aged over 5 years (82%).

We asked the participants for their opinion on the correct time of use of MDs. Referring to children aged 0–2 years, more than half of the sample (62%) indicated “zero minutes” and 36% indicated a maximum of one hour. Particularly, parents of children aged over 5 years (81%) followed by non-parents (64%) declared that children aged 0–2 years should not use MDs.

Referring to older children (3–5 years old), 25% answered that use was not correct (zero minutes). Most of the respondents declared that “less than one hour” is a correct amount of time (66%). Zero minutes were indicated by 45% of parents of children aged over 5 years and 27% of non-parents, compared to 19% of 0–5-year-old children’s parents (*p* < 0.05).

We carried out a multivariate analysis to evaluate the factors associated with perception that the use of a MD by children aged 0–5 years is a risk for their health (Table 3).

After adjusting for age, gender, educational level and number of MDs used, being non-parents (OR 2.3) or having children older than 5 years old (OR 1.8) increased the probability to consider MDs use as a risk for children’s health.

**Figure 1:**
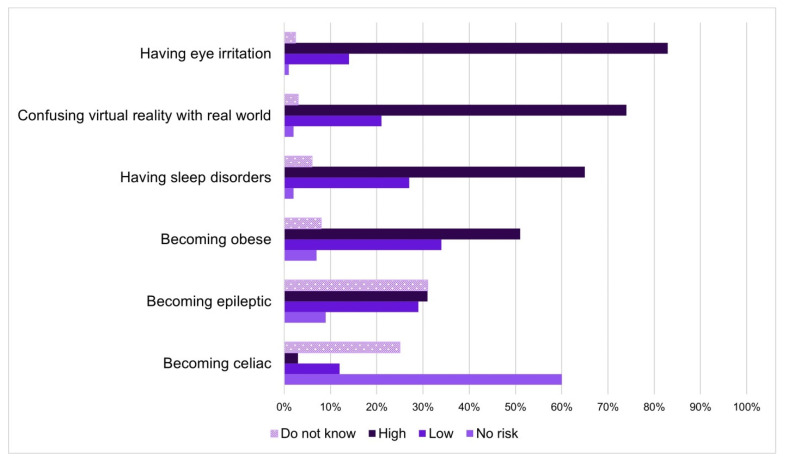
Evaluation on the risk of developing certain situations/symptoms regarding the use of MDs by children under the age of 5, using a 7-point Likert scale. No risk = score 1, Low = scores 2, 3, 4, High = scores 5, 6, 7.

**Figure 2:**
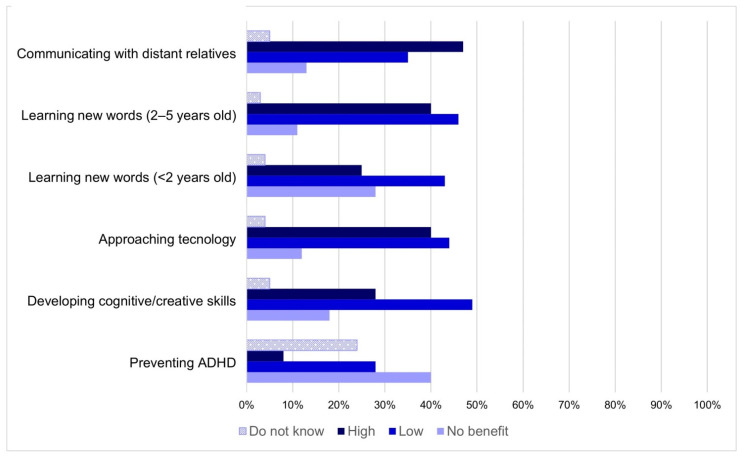
Evaluation of benefits obtainable from certain situations regarding the use of MDs by children under the age of 5, using a 7-point Likert scale. No benefit = score 1, Low = scores 2, 3, 4, High = scores 5, 6, 7.

Considering the whole sample, specific risks and benefits related to the use of MDs by children under the age of 5 have been investigated (Figure 1 and Figure 2).

About risks (Figure 1), having eye irritation (*n* = 2582; 83%), confusing virtual reality with real world (*n* = 2294; 74%) and having sleep disorders (*n* = 2015; 65%) were situations considered most risky, as indicated by respondents. Becoming obese was considered a high risk by 51% (*n* = 1598) and a low risk by 32% (*n* = 981) of respondents. Thirty-one percent (*n* = 956) did not know to quantify the risk of developing epilepsy and the same percentage rated the risk high. Sixty percent of the sample (*n* = 1857) reported that there was no risk related to developing celiac disease with the use of MDs and 36% (*n* = 797) were not able to give a score.

Excluding “becoming epileptic” and “coeliac”, in all the other items, more than 50% of respondents rated high risk.

Communicating with distant relatives (*n* = 1448; 47%) was the situation most perceived as beneficial by participants. All other possible benefits related to the use of MDs were perceived as high by less than 50% of the sample (approaching technology, learning new words, developing cognitive/creative skills). Preventing ADHD (Attention Deficit Hyperactivity Disorder) was not indicated as a benefit by 40% of the sample. However, 25% (*n* = 755) of respondents were not able to give a score. Twenty-eight percent of respondents (*n* = 869) stated that learning new words was not a benefit related to the use of MDs by children under the age of 2 years, and to a greater extent when referring to children aged 3–5 years (11%, *n* = 349) (*p* < 0.001).

The distribution of responses for each risk or benefit did not change substantially when non-parents, 0–5-year-old children’s parents and parents of children over 5 years were considered separately (data not shown in figures).

Questions related to reasons and time of use of MDs by children aged 0–5 years were specifically addressed to parents of children under 5 years. Excluding 32 parents who did not complete this part of the questionnaire, the analysis was carried out on 1869 parents. Of them, 587 parents had only children aged 0–2 years, 994 had only children aged 3–5 years and 288 had children in both age groups.

Fifty-four percent (*n* = 1029) of preschoolers’ parents declared to allow them MDs use. Parents reported an average use time of 61 ± 40 min.

More than half (56%) declared that their children became annoyed or tried to oppose when it is time to stop using MDs. This happened more among parents who allowed the use of MDs to their children (62% vs. 49%; *p* < 0.001).

Parents who let their children use the mobile phone were also asked if children used it alone. Autonomous use occurred in about half of the cases (*n* = 549; 54%).

It may happen that the child himself asks to use MDs. Parents admitted that this occurred in 82% of cases (*n* = 1561). The main reasons for this request were attributable to watching generic videos (*n* = 934; 60%), watching cartoons (*n* = 643; 41%), playing games (*n* = 619; 40%), taking photos (*n* = 473; 30%) and listening to music or looking at photos (*n* = 112; 7%).

Reasons why parents offered MDs to their children were to distract them (*n* = 372; 40%), to keep them calm (*n* = 252; 27%) and to communicate with relatives/friends (*n* = 104; 11%). Other reasons given were educational purposes (*n* = 83; 9%) and fun (*n* = 47; 5%). When it came time to quit using MDs, parents reported that in about half of the cases, children become annoyed and/or try to oppose (*n* = 1051; 56%).

We analyzed the responses on the correct use of MDs by children aged 0–2 and 3–5 years in relation to actual use, respectively in parents with only children aged 0–2 years (*n* = 587) and parents with children aged 3–5 years, or children belonging to both age groups (*n* = 1282).

A separate analysis for parents who have exclusively children aged 3–5 years and children belonging to both ages (0–2 and 3–5 years) was carried out without finding significant differences.

**Figure 3:**
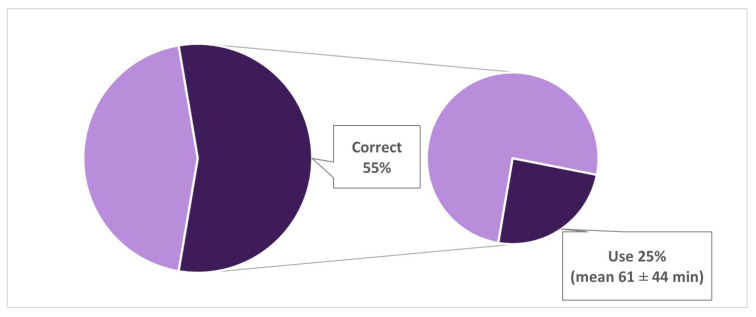
Comparison between opinions about the correct time of use by children aged 0–2 years and responses about the actual use (Correct answer = zero minutes) [21]. Analyses limited to parents having only children aged 0–2 years.

Fifty-five percent (*n* = 306) of parents with only children aged 0–2 years declared that it was not correct to use MDs with toddlers, however 25% of them (*n* = 75) allowed their children to use MDs. The mean time of use reported was 61 ± 44 min, and the use alone occurred in 48% of cases (Figure 3). Overall, 37% (*n* = 214) of parents with children aged 0–2 years declared to allow the use of MD by their children, and alone in 51% (*n* = 109) of cases.

**Figure 4:**
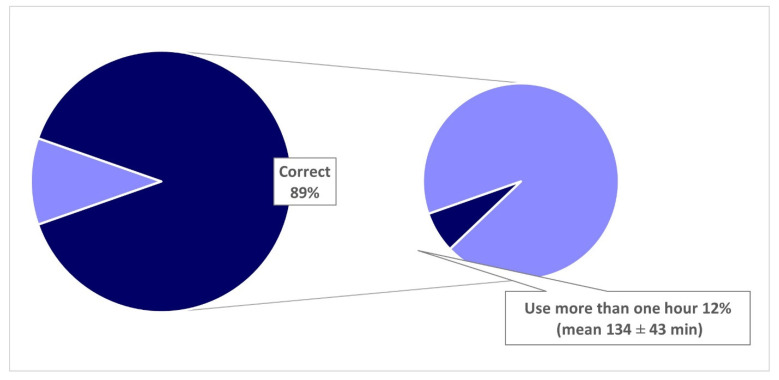
Comparison between opinions about the correct time of use by children aged 3–5 years and responses about the actual use (Correct = one hour maximum) [21]. Analyses limited to parents having only children aged 3–5 years or both children aged 0–2 and 3–5 years.

With regard to MDs use in children aged 3–5 years, 89% (*n* = 1083) of parents with children aged 3–5 years or children belonging to both age groups indicated that it was correct to allow use of MDs for up to a maximum of one hour a day, and 12% of them allow their children to use it for more than an hour (Figure 4). The mean time of use reported was 134 ± 43 min, and the use alone occurred in 78% of cases. Overall, 62% (*n* = 796) of this group of parents declared to allow the use of MD by their children, and alone in 54% (*n* = 426) of cases.

Twenty-eight percent of parents with children aged 0–2 years who thought that using MDs is a risk for children’s health let their children use MDs. The use was also allowed by the 71% of those who did not think that there is a risk and the 61% of those who did not know (*p* < 0.001). Among parents having only children aged 3–5 years or children belonging to both age groups (0–2 and 3–5 years), the use of MDs was admitted by the 60% of parents claiming that there is a risk, the 87% of those who declared that there is no risk and finally, the 82% of those who did not know (*p* < 0.001).

In order to understand the characteristics of parents most inclined to allow use of MDs to their children, the variable concerning the question “Do you usually let your child use mobile devices?” was used as a dependent variable in the multivariate logistic regression analysis. Results showed that a low level of education (high school or lower) (OR 1.77, 95%CI: 1.40–2.24), having more than one child (OR 1.58, 95%CI: 1.25–2.01), using more than one MD (OR 1.57, 95%CI: 1.21–2.04) and perceiving low risk (OR 2.23, 95%CI: 1.71–2.91) were predictors of easier permission of MDs use by parents. Odds ratios have been adjusted for age and gender.

About half of the preschoolers’ parents received or sought information on risks related to MDs’ use (54%; *n* = 1011). The most consulted sources of information were, in order of prevalence (multiple choice), web (78%; *n* = 787), pediatrician (22%, *n* = 221) and municipal or school meetings (15%; *n* = 147).

## 4. Discussion

This survey analyzed the overall perceptions about use of MDs in children, especially in the preschool age, and the relationship between parents’ risk perception and attitude to MDs use by their children.

Most of the respondents considered that MDs use could be harmful for 0–5-year-old children’s health. In order to better interpret the overall risk perception, participants were asked to rate specific risks and benefits related to MDs use. A high prevalence ranging from 60% to 80% of participants indicated sleep disturbances and developing eye irritation as situations related to risk. In fact, continuous eye exposure to close screens causes faster evaporation of the tear film, which may lead to dry eye disease and ocular fatigue [19]. The same goes for sleep quality, where evidence supports a correlation between any form of screen-time (MDs, television and computer) and poor sleep outcome (delayed bedtime, shortened total sleep time, daytime tiredness and sleep-onset latency) [16]. Evidence specifically relating to the 0–5-year-old group is still poor but supports these findings. The risk of confusing virtual reality with the real world is also perceived as a high risk in most cases (74%). In fact, children’s learning is subordinated to direct experience in the real world, as described by Bozzola and co-authors, who claimed that in this age group, infants’ and toddlers’ touch screen usage might interfere with learning development [21]. They need direct first-hand experience with materials and equipment that challenge their thinking and problem-solving skills [31].

Risk of developing obesity, demonstrated by the scientific literature in relation to sedentary lifestyle [32,33], has been rated to a lesser extent than previous items, probably because obesity was perceived as an indirect event and therefore less concrete and temporally more distant than eye irritation or sleep problems.

Anyway, overall risks were perceived more than possible benefits. In fact, for each investigated benefit, less than half of the interviewees rated high scores. This was consistent with the result of high risk perceptions in general. Where MDs use includes sharing with an adult and interactivity, digital technology can be taken as an opportunity for children’s learning [34,35]. However, the possibility of learning new words was rated “low” as a benefit by interviewees in most cases, particularly for children aged less than 2 years. These responses are consistent with the guidelines of the American Academy of Pediatric Children (AAP) [22], which indicate that there is an opportunity to learn through the use of MDs, but children learn better when they are re-taught in the real world than when they learn through a screen.

Both among risks and benefits, items for which there was no scientific evidence on association with MD use (i.e., ADHD, celiac disease, and epilepsy) were rated with low scores, as expected and therefore confirming the appropriateness of the responses.

Based on protection motivation theory [36], we hypothesized that parents of 0–5-year-old children, who are usually more apprehensive about the health of their children, especially if they are very young, would have perceived the MDs’ use risk to be greater than others.

Contrary to what was assumed, risk appeared to be perceived more by non-parents and parents of older children compared to parents of 0–5-year-old children. It was confirmed that risk perception should be considered from a number of perspectives. A qualitative study across Europe showed that, regardless of the position taken by parents in relation to risks and benefits of MDs, the fact that their use is unavoidable for the children dominates parents’ viewpoints [15].

In fact, smartphones and tablets have become tools constantly present in daily life of children and as often happens, habit can diminish the perception of risk. For those who do not have children, the perceived risk is more theoretical. The reason why parents of older children also perceive a higher risk than parents of younger children may be attributed to the fact that in older children, possible negative effects of MDs use could become evident.

The perception may also be influenced by a greater difficulty in managing children’s screen time as they grow up [37,38]. Parents of younger children might therefore perceive a lower risk because they think to be able to better control their children’s behavior and protect them from possible health risks.

Independently from parental status, the perception that MD use was harmful for children’s health was associated with increasing age of participants, high education level and being female.

Similarly, when respondents were asked their opinion about the correct time of use of MDs by children aged 0–2 and 3–5 years, parents of children over 5 years old were found to be less permissive than parents of children aged 0–5 years and non-parents.

Overall, answers provided by interviewees regarding the time of use of MDs according to guidelines were satisfactory and consistent with questions investigating perception of risk. In fact, with reference to children aged 3–5 years, for whom guidelines allowed use up to one hour a day, most of the sample (91%) responded correctly. Also, in case of children aged under 2 years, most of the sample thought correctly that the time of use is zero, even if mainly parents of older children and non-parents.

About half of the parents reported granting MDs use to their 0–5-year-old children, as also shown elsewhere [10,37]. Taking into account SIP guidelines [21], we tried to understand how much perception influenced behavior. Just over half of the parents of children aged under 2 years responded that MDs should not be used by toddlers, however a quarter of them allowed MDs use, for an average daily time of about an hour. Less evident was the discrepancy between theory and practice for parents who had at least one child aged 3–5 years old. They almost all reported that use of up to one hour a day was correct, even if about one in ten parents reported to allow the MDs use to their children for a much longer time. These data seem to support that the knowledge of the correct behavior does not directly translate into avoiding MDs use as recommended.

This discrepancy confirmed that risk perception was not the only determinant of proper behavior. Previous studies on obesity prevention in children showed that difficulties of parents to put into practice healthy behavior awareness and knowledge were attributed to failure to barriers such as lack of time and pressure from their children [29,30]. Of course, living reality is different from imagining it. Facing the reality for parents means to deal with children crying or having a tantrum when you have so many things to do. In this study, we found that most parents who declared that children tried to oppose when it was time to stop using MD allowed their children to use it. In fact, it was shown that parental control mediated by parental self-efficacy was associated with lower levels of screen viewing among children aged 5–6 years old [13]. Another study focusing on screen time and eye care behavior of children [14] highlighted that parental mediation of smartphone use by children could depend on perception of the severity of the problem in addition to self-efficacy.

According to other published findings [39], the multivariate analysis showed that having more than one child seemed to be a predictive factor to allow children to use MDs, probably because use increases with children growing and also because having more than one child implies the need for “help” in babysitting. Moreover, older children are more likely to use mobile screen media devices compared with their younger counterparts and younger children usually tend to imitate older brothers/sisters.

Using more than one MD was inversely associated with the opinion that MD use was harmful for children’s health, and analyzing only parents of 0–5-year-old children, this factor was associated with allowing children to use MDs. In fact, the findings of a recent review demonstrated that parental behaviors and home environment could be more influential in shaping children’s behavior [20], and it was clearly shown that parents who spend more time using computers and MDs had children who spend more time using MDs [10,25,26,40].

By investigating the main reasons why MDs were given to children, our study showed that they were related to MDs role of “pacifiers”. In more than half of the cases, these were allowed to children to calm or distract them, contrary to what was recommended in the guidelines [21]. The parent–child bond is a great resource in containing the child’s crying/discomfort moments. Using MDs as a substitute for this type of bond will negatively limit the child’s control of his/her emotions.

Half of parents declared to allow their children to use MDs alone, both in case of children aged 0–2 and 3–5 years, as also shown elsewhere [21,38]. This could expose children to inappropriate content and unsupervised use time in addition to promote disconnection from the surrounding context. Some authors highlighted that the massive penetration of MDs influenced communication between parents and children so much that it was becoming more and more common to see parents and children together but absorbed by the screen instead of being attuned to each other [13,41].

It is therefore essential that children share the use of MDs with adults to promote learning and interactions [35]. It is interesting to note that co-using MDs with parents has been shown to reduce conflict more than restrictive mediations [42].

Recently, AAP published the “Family Media Plan”, a personalized plan that allows parent-users to think about media, and create goals and rules that are in line with family context. This tool was created to prevent media from replacing many other important activities, such as face-to-face interaction, family time, outdoor play, exercise, unplugged downtime and sleep [43].

It is recommended to designate media-free times together, such as dinner or driving, as well as media-free locations at home, such as bedrooms. AAP generally recommend parents to be a good role model, because children are great mimics: they learn above all by example, rather than by verbal teaching. Regarding the role of “emotional pacifier”, AAP explains that media can be very effective in keeping kids calm and quiet, but it should not be the only way they learn to calm down. They need guidance to manage strong emotions, like talking about ways to solve the problem or, for younger children, to channel emotions.

When source of information was investigated, respondents indicated mainly the web (78%), and to a lesser extent, reliable sources such as the pediatrician (22%). This finding evidenced that the topic was also relatively new among pediatricians, despite the presence of guidelines. It was shown that only 16% of pediatricians ask families how much they use MDs and only 29% of parents reported that they have asked their pediatrician for advice [31]. However, it is clear that the role of pediatricians in warning about MDs exposure in childhood is necessary [21]. Since the web is widely used as a source of information, this could become a resource if parents were given guidance on how to search and use the most trusted websites. Also, in this sense, pediatricians and health professionals could all play a more important role.

Some limitations should be addressed. The main limitation, typical of the online survey, is that participants were a convenience sample and therefore not representative of the general population. Moreover, it would be interesting to investigate knowledge and risk perception of other figures who increasingly interact with preschool children, for example grandparents, uncles and babysitters. Another limitation is that data were self-reported and may possibly be affected by some inaccuracies, mainly when parents were asking about type and time of use of MD by their children. However, it is likely that the real use was underestimated considering that high perceived risk may have influenced the responses regarding the use of MDs.

## 5. Conclusions

There was an overall high perception of risk associated with the use of MDs by children and awareness about a proper screen time use. Despite this, it seemed that children were allowed to use MDs too early, even in the age groups in which guidelines prohibit use and, if this was allowed, for too long. Furthermore, a discrepancy between risk perception and permissive behavior in 0–5-year-old children’s parents was evidenced. This could be due to the fact that the association between MD use and health risks was unclear, considering that the scientific evidence on this issue is still poor, mainly in younger children. In fact, the pediatrician himself was not the main source of information about it. Moreover, digital tools have become so pervasive that it was shown that the inevitability of the issue dominated the views of parents [15]. This means that daily routines that include doing household chores, going to work, managing a crying baby and so on could overlook the risk perception.

Unlike other pediatric associations [21,22], the Royal College of Pediatrics and Child Health in the UK suggested that parents have to impose limits when they realize that excessive use of MDs produces negative effects in the child (sleep loss, interference with family or school activities, etc.) [24]. Furthermore, they emphasized the role of the parent as an example for the child thinking about their own use. Children are great imitators, and therefore adults must be the first to review their own ways of using MDs [24,42].

To this purpose, increasing knowledge on what are the determinants of parents’ perception and behavior is needed to design effective and tailored family-based interventions in order to guide parents to moderate and more careful use of MDs by their children and improve child–parent interaction. This means on the one hand providing advice on alternatives to MDs such as games, physical activity and other sharing activities, and on the other hand, to take advantage of digital technologies’ possible benefits, using MDs with and not just next to the child.

In this context, health professionals and particularly pediatricians need to be more present also through the web, which has been confirmed as one of the major sources of information to raise awareness among the general population on this issue (not only parents, but also other caregivers such as grandparents, babysitters, etc.). It is certainly important to pay more attention to this issue considering that only half of parents were informed or felt the need to do so.

## Figures and Tables

**Table 1:** Socio-demographic characteristics of participants.

Characteristics	*n*	%
Gender		
Female	2689	86
Male	426	14
Age		
18–35 years	1615	52
>35 years	1500	48
Nationality		
Italian	2957	95
Other	158	5
Educational level		
High school or less	2009	65
University degree	1106	36
Employment status		
Student	189	6
Housewives	499	16
Employee worker	1738	56
Self-employed	427	14
Retired	78	2
Unemployed	184	6
Parents	2540	82
Mother	2295	90
Father	245	10
Parents of 0–5-year-old children	1901	75
Number of children		
One child	1103	58
More than one child	789	42

**Table 2:** Comparison of parents and non-parents regarding risk perception and opinions on mobile devices (MDs) use by children aged 0–5 years.

Questions	All Participants *N* (%)	Parents of Children Aged 0–5 Years (A) N (%)	Parents of Children Aged > 5 Years (B) N (%)	Non-Parents (C) N (%)
Does the use of a MD by a child (0–5 years) pose a risk to his health? (n = 3115)				
Yes	2304 (74)	1325 (70) ^1,2^	519 (81)	460 (80)
No	282 (9)	191 (10)	46 (7)	45 (8)
Don’t know	73 (17)	385 (20)	73 (11)	70 (12)
How harmful do you think MD use is on a scale of 1 = not at all to 7 = very much? (n = 2304)				
Score ≥ 5	1758 (76)	1003 (76) ^1^	424 (82) ^3^	331 (72)
Score ≤ 4	546 (24)	323 (24)	96 (18)	127 (28)
How long do you think it is correct that a child aged 0–2 years use a MD per day? (n = 2855) ^a^				
Zero min	1757 (62)	974 (55) ^1,2^	455 (81) ^3^	328 (64)
Until 60 min	1034 (36)	764 (42)	99 (18)	171 (33)
More than 60 min	64 (2)	46 (3)	5 (1)	13 (3)
How long do you think it is correct that a child aged 3–5 years use a MD per day? (n = 2860) ^b^				
Zero min	722 (25)	335 (19) ^1,2^	247 (45) ^3^	140 (27)
Until 60 min	1876 (66)	1272 (71)	278 (51)	326 (64)
More than 60 min	262 (9)	196 (10)	19 (4)	47 (9)

MD = Mobile Device. ^1^ A vs. B; *p* < 0.05; ^2^ A vs. C; *p* < 0.05; ^3^ B vs. C; *p* < 0.05. ^a^: Five respondents answered “I don’t Know” and they were excluded. ^b^ N = 255 responses were missing.

**Table 3:** Factors associated with perception that the use of a mobile device by children aged 0–5 years is a risk for their health.

Variables	Adjusted OR	95% Confidence Interval	*p*-Value
Age (risk increase per year)	1.01	1.0–1.03	0.007
Gender			
Female vs. Male	1.53	1.18–1.97	0.001
Educational level			
University degree vs. High school or less	1.22	1.02–1.46	0.03
Number of MDs used			
One MD vs. More than one MD	1.21	1.01–1.44	0.04
Parenting			
Having children aged 0–5 years	Ref		
Having children aged > 5 years	1.84	1.38–2.44	<0.001
No children	2.29	1.77–2.96	<0.001

## Data Availability

The data presented in this study are available on request from the corresponding author.

## Supplementary Materials

The following are available online at https://www.mdpi.com/article/10.3390/ijerph18073440/s1, Supplementary_File S1: Questionnare.
