# Supplementary material for: Parents and Mobile Devices, from Theory to Practice: Comparison between Perception and Attitudes to 0–5 Year Old Children’s Use
Source: Int J Environ Res Public Health. 2021 Mar 26;18(7):3440. doi: 10.3390/ijerph18073440 (PMC8037713; doi:10.3390/ijerph18073440)
Supplement: Supplementary file 1 [file ijerph-18-03440-s001.pdf]

Supplementary file: Questionnaire

A: Sociodemographic characteristics

1. Do you agree to participate in this research?
    - o Yes
    - o No
2. Gender
    - o M
    - o F
3. Age ______

4. Educational qualification
    - o Primary school certificate
    - o Secondary school certificate
    - o High school diploma
    - o University Degree / Master
5. Employment
    - o Student
    - o Housewife
    - o Employee
    - o Self-employed
    - o Retired
    - o Unemployed
6. Country of origin
    - o Italy
    - o Other _____

B. Questions addressed to all participants

7. How many Mobile Digital Devices (smartphone or tablet) do you use every day?
    - o None
    - o 1
    - o 2
    - o 3
    - o More than 3
8. How many hours do you spend, overall, in front of your smartphone / tablet during the day? __________

9. In your opinion, does the use of smartphones and / or tablets by a preschooler (0-5 years old), pose a risk to his/her health?
    - o Yes
    - o No
    - o I don't know

10. If yes, how harmful do you think it is on a scale of 1 to 7? *(1 = not harmful; 7 = very much harmful)*

□ 1 □ 2 □ 3 □ 4 □ 5 □ 6 □ 7

11. How long do you think it is correct the use of a smartphone and / or tablet, during the day, for:
    - A child aged under 2 year.
        - __________(minutes)
        - I don't know
    - A child aged 3-5 years old.
        - __________(minutes)
        - I don't know

12. Referring to the use of smartphone and / or tablet by a preschooler, how much is, in your opinion, the risk of:
    (Scale of 1 to 7; 1 = not at all risky; 7 = extremely risky*)*
    - Having sleep disorders

      □ 1 □ 2 □ 3 □ 4 □ 5 □ 6 □ 7 □ I don't know

    - Becoming obese

      □ 1 □ 2 □ 3 □ 4 □ 5 □ 6 □ 7 □ I don't know

    - Becoming epileptic

      □ 1 □ 2 □ 3 □ 4 □ 5 □ 6 □ 7 □ I don't know

    - Having eye irritation

      □ 1 □ 2 □ 3 □ 4 □ 5 □ 6 □ 7 □ I don't know

    - Becoming celiac

      □ 1 □ 2 □ 3 □ 4 □ 5 □ 6 □ 7 □ I don't know

    - Confusing virtual reality with the real world

      □ 1 □ 2 □ 3 □ 4 □ 5 □ 6 □ 7 □ I don't know

13. Referring to the use of smartphone and / or tablet by a preschooler, how much is, in your opinion, the risk of:
    (Scale of 1 to 7; 1 = no benefit at all; 7 = high benefit)
    - Learning new words (for children aged under 2 years old)

      □ 1 □ 2 □ 3 □ 4 □ 5 □ 6 □ 7 □ I don't know

    - Learning new words (for children aged 3-5 years old)

      □ 1 □ 2 □ 3 □ 4 □ 5 □ 6 □ 7 □ I don't know

    - Developing cognitive/creative skills

□ 1 □ 2 □ 3 □ 4 □ 5 □ 6 □ 7 □ I don't know

- Preventing ADHD (Attention Deficit and Hyperactivity Disorder)

  □ 1 □ 2 □ 3 □ 4 □ 5 □ 6 □ 7 □ I don't know

- Communicating with distant relatives

  □ 1 □ 2 □ 3 □ 4 □ 5 □ 6 □ 7 □ I don't know

- Approaching technology

  □ 1 □ 2 □ 3 □ 4 □ 5 □ 6 □ 7 □ I don't know

14. Do you have children?
    - No
    - Yes

C. Questions addressed to preschoolers' parents

15. If yes, do you have preschool children (0-5 years old)?
    - No
    - Yes
16. If yes, how old are they?

    Child 1 _____

    Child 2 ____

    Child 3 ____

    Etc.

17. Do you usually let your child use the smartphone and / or tablet?
    - Yes
    - No
18. If yes, how long per day? _________________
19. Do you let him/her use it alone?
    - Yes
    - No
20. If yes, how long do you let him use alone?
    - Less than half the time
    - About half the time
    - More than half the time
21. Has the child ever requested the device from you, if he is able to?
    - Yes
    - No
22. If yes, for what reason? *(More than one answer allowed)*
    - Playing
    - Using educational apps
    - Taking pictures

- Watching videos
- Watching cartoons / TV series
- Other _______________

23. When it is time to stop using your smartphone or tablet, does your child annoys and try to oppose?
    - Yes
    - No
24. If you give your child the smartphone and / or tablet, what is the main reason? *(Only one answer)*
    - To make him/her feel good
    - To make him/her stop crying
    - Because it favors his/her cognitive development
    - To approach him/her to technology
    - To distract him/her while I do cleaning, work, focus on other things
    - To communicate with relatives / friends (e.g. videochat)
    - Other _______________
25. Have you ever sought or received information about risks associated with using smartphone / tablet?
    - Yes
    - No
26. If yes, what was the source of information? *(More than one answer allowed)*
    - Website/social media
    - Meetings organized by Municipality
    - Meetings organized by Schools
    - Pediatrician
    - Other _________________________

N.B. The original questionnaire was in Italian language.
